# Epidemiologic measures and policy formulation: lessons from potential outcomes

**DOI:** 10.1186/1742-7622-2-5

**Published:** 2005-05-27

**Authors:** Sander Greenland

**Affiliations:** 1 Departments of Epidemiology and Statistics, University of California, Los Angeles, USA

## Abstract

This paper provides a critique of the common practice in the health-policy literature of focusing on hypothetical outcome removal at the expense of intervention analysis. The paper begins with an introduction to measures of causal effects within the potential-outcomes framework, focusing on underlying conceptual models, definitions and drawbacks of special relevance to policy formulation based on epidemiologic data. It is argued that, for policy purposes, one should analyze intervention effects within a multivariate-outcome framework to capture the impact of major sources of morbidity and mortality. This framework can clarify what is captured and missed by summary measures of population health, and shows that the concept of summary measure can and should be extended to multidimensional indices.

## Introduction

This paper describes a set of fundamental concepts from causality theory that can be used to critically analyze summary measures of population health. It then uses these concepts to argue that health measures based on hypothetical outcome removal [1,2] are ambiguous and potentially misleading. Thorough analyses require a multivariate framework to capture what is known about sources of morbidity and mortality. Because of the unavoidable shortcomings of single-valued summaries, multidimensional indices should be considered for summary measures of population health.

The first task is to define cause and effect in a manner precise enough for logical manipulation and quantification. Three types of models have achieved widespread usage:

• *Counterfactual or potential-outcome models*. The counterfactual conceptualization of causality was glimpsed by Hume [3] and was operationalized by statisticians in the 1920s and 1930s in the form of potential-outcome models [4]. Related counterfactual models have also received much attention in philosophy [5,6], and potential outcome models are now common in social sciences [7,8] and epidemiology (see citations below) as well as in statistics.

• *Structural-equations models*. These models can be traced to early work in path analysis in the 1920s and were given full expression by econometricians in the 1940s.

• *Graphical models *(causal diagrams). These models also originated in path analysis, but were not fully developed until the early 1990s [9-11].

In his book on causal theory, Pearl [11] details the above approaches and their histories, emphasizing that they are logically equivalent (isomorphic) for all practical purposes. This means that an analysis using one approach can be translated into either of the other two approaches, while maintaining logical consistency. Greenland and Poole [12] and Greenland and Brumback [13] describe the connections between potential outcome models and the sufficient-component cause models familiar to epidemiologists, and Greenland [14] discusses potential outcome models in relation to more traditional approaches to causal inference.

Because of the emphasis on counterfactual models in the literature on measures of cause and effect, the following development is based on them. Further details of counterfactual theory for health science research are described elsewhere [12,14-26]. For details on its relation to missing-data models, see Rubin [27]. The present paper concerns only issues of defining effects and their implications for policy formulation. Equally important is quantitative accounting for study biases in estimating effects; see Greenland [28,29], Lash and Fink [30], and Phillips [31] for recent developments in that topic.

## Basic concepts of counterfactual causality

### Actions, outcomes and counterfactuals

To minimize ambiguity, a counterfactual model requires reasonably precise definitions of the following model ingredients:

• At least one *target *subject of interest in whom or which causation is to be studied (e.g. a specific person or population).

• A list of two or more possible alternative actions, *x*_0_, *x*_1_, etc., that could have been applied at or over a span of time, one of which may be the actual action taken.

• An outcome measure, *Y*, taken at a point in time or over a period of time, following completion of the action.

As an example, the subject could be Kosovo, the action *x*_1_could be revocation of its autonomy by Yugoslavia in 1988, an alternative *x*_0 _could be that revocation never occurred, and the outcome measure could be the mortality rate of pre-1999 residents during 1999. Because only one of the possible actions *x*_0_, *x*_1_, etc. can take place, all but one of the actions must become *counterfactual*, or contrary to fact. In the example, the actual (or factual) action was *x*_1 _= "revocation of autonomy"; thus, the action *x*_0 _= "no revocation" is counterfactual. (This example also illustrates the truism that to do nothing is an action, corresponding to zero on an action scale.)

If *x*_*a *_is the actual action taken (from the list *x*_0_, *x*_1_, etc.), we may observe the outcome *Y*(*x*_*a*_) that follows that action. A *counterfactual outcome model *posits that, for any counterfactual action, *x*_*c *_(from the same list), there is also a well-defined outcome, *Y*(*x*_*c*_), that would have followed *that *action. The entire list of such outcomes *Y*(*x*_0_), *Y*(*x*_1_), *Y*(*x*_2_), ... is called the set of *potential outcomes *of the subject; it includes both the actual outcome *Y*(*x*_*a*_) as well as all the outcomes of counterfactual actions.

In the example, the actual action, *x*_*a*_, was *x*_1 _and was eventually followed by a mortality rate, *Y*(*x*_1_), in 1999. *Y*(*x*_1_) is difficult to assess but nonetheless exists as a matter of fact; it is part of what occurred subsequently to the action *x*_1_. A counterfactual model for 1999 mortality posits that, had the counterfactual action *x*_0 _been taken instead (that is, if no revocation had occurred), the mortality would have equaled some number, *Y*(*x*_0_). Given that *x*_0 _is counterfactual, it is logically impossible for us to observe *Y*(*x*_0_). Nonetheless, the counterfactual model treats this number as a precise, though unknown, quantity.

The idea of treating the outcome, *Y*(*x*_*c*_), under a counterfactual action, *x*_*c*_, as a precise quantity has been a source of much controversy and misunderstanding (e.g. [23,32,33]). Some major misunderstandings are addressed below:

1) The counterfactual approach does *not *require that the outcome, *Y*(*x*_*c*_), be precisely defined for every possible counterfactual action, *x*_*c*_. In the above example, if we are interested only in contrasting revocation (*x*_1_) with no action (*x*_0_), our model need not mention any other actions. That is, we can limit the action list to just those actions of interest.

2) The counterfactual approach is *not *inherently deterministic in either the classical or quantum-mechanical sense [15,34]. The potential outcomes, *Y*(*x*_0_), *Y*(*x*_1_), etc., may represent different values for a statistical parameter in a classical probability model. For example, they may be expected rates in Poisson models. Alternatively, they may represent different mixtures of superposed states (different wave functions) in quantum models. Indeed, some theoreticians regard counterfactuals as essential for formulating coherent explanations of quantum phenomena [35].

3) The counterfactual approach extends the partial quantification of outcomes, *Y*(*x*_*c*_), under counterfactual actions embedded in ordinary discourse. In the example, some (though not all) observers of the events in Kosovo 1988–1999 speculated that the actual 1999 mortality, *Y*(*x*_1_), was probably greater than *Y*(*x*_0_), the mortality that would have occurred had autonomy never been revoked. This speculation arises from the following tentative explanation of actual events in Kosovo: revocation of autonomy, (*x*_1_), caused Albanian resistance to increased Serb authority, which in turn caused Serbian leaders to extend their "ethnic cleansing" policy to Kosovo. Had there been no revocation, (*x*_0_), this tragic causal sequence of events would not have occurred.

### Cause and effect

The speculative explanation in the third bulleted item above is an example of an informal causal hypothesis. Consideration of such hypotheses has led to the following definition: An *effect *of taking an action, *x*_*j*_, rather than another action, *x*_*k*_, on an outcome measure, *Y*, is a numerical contrast of that measure (e.g. the difference or ratio) under the two different actions. The contrast is called an *effect measure*. In the example, the contrast *Y*(*x*_1_) - *Y*(*x*_0_) is an effect of revocation *x*_1 _versus no revocation *x*_0_; this effect measure is known as the mortality difference due to x_1 _versus *x*_0_. Similarly, *Y*(*x*_1_) / *Y*(*x*_0_) is the effect measure known as the mortality ratio due to x_1 _versus *x*_0_.

Many common ideas and a few surprises follow from the above definitions, among them:

4) An effect is a *relation *between the outcomes that would follow *two *different actions, *x*_*j *_and *x*_*k*_, in just *one *subject (a population or single person). It is thus meaningless to talk of (say) "the effect of smoking a pack a day"; one must at least imply a reference (baseline) action for "the effect" to have meaning. While smoking a pack a day can cause lung cancer relative to no smoking, it can also prevent lung cancer relative to smoking two packs a day.

5) If *Y*(*x*_*j*_) = *Y*(*x*_*k*_), we say that having *x*_*j *_happen rather than *x*_*k *_had no effect on *Y *for the subject; otherwise, we say that having *x*_*j *_happen rather than *x*_*k *_caused the outcome to be *Y*(*x*_*j*_) and prevented the outcome from being *Y*(*x*_*k*_). For example, we may say that smoking prevents survival past age 70 years just as surely as it causes death by age 70. Similarly, we may say that not smoking causes survival past age 70 just as surely as it prevents death by age 70. Thus, the distinction between causation and prevention is merely a matter of whether we are talking of an action, *x*_*j*_, and *its *consequence, *Y*(*x*_*j*_) (causation of *Y*(*x*_*j*_)), or an action, *x*_*j*_, and a consequence, *Y*(*x*_*k*_), of an alternative action *x*_*k *_≠ *x*_*j*_(prevention of *Y*(*x*_*k*_)).

6) At least one of the actions, *x*_*j*_, *x*_*k*_, in an effect measure must be counterfactual. Thus, we can never observe an effect measure separate from an outcome measure. In the example, we observed the mortality *Y*(*x*_1_) = *Y*(*x*_0_) + [*Y*(*x*_1_) - *Y*(*x*_0_)], so the mortality difference, *Y*(*x*_1_) - *Y*(*x*_0_), is mixed with the reference (baseline) mortality rate, *Y*(*x*_0_), in our observation. The best we can do is make an informed estimate of *Y*(*x*_0_), which is the outcome that would have happened under the counterfactual action *x*_0_, and from that estimate deduce an estimate of the effect measure (by subtraction, in this example).

### Causation, confounding and association

Problem 6 is considered a fundamental problem of all causal inference. It was recognized by Hume [36] and is now known as the *identification problem *of cause and effect. All causal inferences (and hence all intervention plans) depend on accuracy in estimating or predicting at least one unobserved potential outcome following one counterfactual action. We ordinarily make this prediction based on observations of other subjects (controls) who experienced actual actions different from the subject of interest. For example, we might estimate that the mortality Kosovo would have experienced in 1999 had there been no revocation, *Y*(*x*_0_), would equal the mortality it experienced in 1988, before violence began growing. In making this estimate, we run the risk of error because, *even under the action *x_0 _(no revocation), Kosovo mortality might have changed between 1988 and 1999. If so, we say that our estimate is *confounded *by this unknown change.

Denote by *Y*_1988 _the mortality experienced by Kosovo in 1988. We can then restate the last problem as follows: we do not observe *Y*(*x*_0_), so we cannot directly compute a measure of the effect of *x*_1 _versus *x*_0_. If, however, we believed the speculative explanation given in the above third bulleted item, we might also think that *Y*_1988 _is not too far from *Y*(*x*_0_), and so substitute *Y*_1988 _for *Y*(*x*_0_) in our measures. Thus, if we also observe *Y*(*x*_1_), the actual 1999 mortality, we would estimate the effect measure *Y*(*x*_1_) - *Y*(*x*_0_) with the observed mortality difference *Y*(*x*_1_) - *Y*_1988_.

The latter observed difference is called a *measure of association*, because it contrasts two different *subjects *(Kosovo in 1999 versus Kosovo in 1988), rather than one subject under two different *actions *in our list (Kosovo in 1999 under revocation versus Kosovo in 1999 with no revocation). Because of the identification problem (we cannot see *Y*(x_0_)), we must substitute a measure of association for the measure of effect. In this usage, the observed difference will misrepresent the effect measure by an amount equal to the difference of the two:

[*Y*(*x*_1_) - *Y*_1988_] - [*Y*(*x*_1_) - *Y*(*x*_0_)] = *Y*(*x*_0_) - *Y*_1988_

This quantity measures the amount of *confounding *in the association measure (the observed difference) when it is used as a substitute for the effect measure. Like the effect measure itself, the confounding measure contains the unobserved *Y*(*x*_0_) and so can only be estimated, not observed directly. Suppose, however, that we know of reasons why *Y*(*x*_0_) and *Y*_1988 _would differ, such as changes in age structure over time. We can then attempt to adjust *Y*_1988 _for these suspected differences, in the hopes of getting closer to *Y*(*x*_0_). *Standardization *is probably simplest example of such adjustment [18,26].

The presumption underlying use of an adjusted effect measure is that it accounts for all important differences between the unobserved (counterfactual) reference outcome, *Y*(*x*_0_), and the substitute, *Y*_1988_, in the above example. The presumption is debatable in most applications; for example, some would argue that "ethnic cleansing" would have spread to Kosovo even without autonomy revocation and Albanian resistance. This problem of uncontrolled confounding is but one of many methodological problems in estimating effects that are discussed in textbooks (e.g. [26]).

## The effects of outcome removal

Consider a question that asks about the health burden attributable to *y*_1 _versus *y*_0_, where *y*_1_and *y*_0_are not actions in the earlier sense, but are themselves alternative *outcomes *such as AIDS death and CHD death. For example, *y*_1 _could be "subject dies of lung cancer" and *y*_0_could be "subject does not die of lung cancer". As in the earlier framework, these outcomes are mutually exclusive possibilities for just one subject at any one time; hence, at least one must become counterfactual. Because they are not interventions, however, there is severe ambiguity in any definition of another outcome, *T*, as a function of the potential outcomes, *y*_1 _and *y*_0_, because *T *depends in a critical fashion on *how y*_1 _and *y*_0 _are caused.

To see this, suppose *T *is years of life lived beyond age 50 (which is age at death minus 50). How would one have brought about *y*_0 _(prevented the lung-cancer death) if the subject were a male lifelong heavy smoker who developed lung cancer at age 51 and died from it at age 54 (and so had *T*(*y*_1_) = 4 years of life after age 50)? If *y*_0 _had been achieved by convincing the subject to never start smoking, *T*(*y*_0_) could be much larger than *T*(*y*_1_), because the risks of many other causes of death (e.g. CHD) would have been much lower as a consequence of never smoking. But if *y*_0 _had been achieved via an unusually successful new chemotherapy for lung tumors, *T*(*y*_0_) might be little changed from *T*(*y*_1_). This would occur if, shortly after remission, the subject had a fatal myocardial infarction whose occurrence was traceable to smoking-induced coronary stenosis.

The problem just described has long been recognized in discussions of estimating the impact of "cause removal" or "removal of competing risks" when the "causes" or "risks" at issue are outcomes rather than actions or treatments [37]. These outcomes are not subject to direct manipulation independent of the earlier history of the subject. Therefore, any realistic evaluation of the impact of their removal must account for other effects of the *means *of removal.

A similar problem arises in the evaluation of ordinary treatments whenever noncompliance can occur. In general, only advice or prescriptions are under control of the health practitioner; what a patient actually receives is affected not only by advice or prescription, but also by the many complex social and personality factors that influence compliance. This leads to manifold problems in evaluating the effects of received treatment [38], for then the treatment a subject receives is only an outcome, *Y*(*x*_*j*_), of an earlier prescriptive action, *x*_*j*_. In most cases, however, this initial action is unambiguous.

Suppose we could avoid the ambiguity problem by introducing a pair of well-defined alternative actions, *x*_1 _and *x*_0 _such that *x*_1 _causes *y*_1 _and prevents *y*_0_relative to *x*_0_. That is, suppose *y*_1 _will follow *x*_1_, whereas *y*_0 _will follow *x*_0_, so that we have *y*_1 _= *Y*(*x*_1_) and *y*_0 _= *Y*(*x*_0_) with *y*_1 _≠ *y*_0_. We may still face a serious confounding problem in the form of "dependent competing risks". Consider again the heavy smoker who develops lung cancer at age 54, with treatment, *x*_0_, being successful chemotherapy. It could be a mistake to calculate this subject's life expectancy, *T*(*x*_0_), from that of other heavy smokers of the same age and sex who had not developed lung cancer, because such smokers may differ in ways that render them not only less susceptible to smoking-induced lung cancer, but also less susceptible to other smoking-induced cancers (perhaps because they have better DNA-repair mechanisms).

More generally, even if the means of removal is precisely defined, feasible and has no side effects, there is rarely a basis to believe, and often good reason to doubt, that removal of a particular outcome (such as a particular cause of death) would be followed by risks similar to risks among persons who, in the absence of intervention, do not experience the removed outcome [37,39]. Unfortunately, standard statistical procedures for projecting outcomes under cause removal (such as Kaplan-Meier/product limit methods and traditional "cause-deleted" life tables) are based on this similarity assumption.

In view of the problems just described, it is reasonable to conclude the following:

7) Projections of the impact of outcome removal (e.g. removal of a particular ICD9 cause of death [1]), rather than an action that brings about outcome reduction, may not be useful for program planning. Except perhaps in some unusually simple cases (e.g. smallpox eradication), the effects of actions and policies do not correspond to simple cause removal.

8) Even when we have a treatment that specifically and completely prevents an outcome, biased effect estimates are likely if one simply projects the experience of those who naturally lack the outcome onto those who avoid the outcome because of the treatment. Only ongoing follow-up of successfully treated subjects can reliably identify the impact of outcome removal.

Problem 7 implies that summary measures for policy formulation should refer to effects of operationalizable actions (e.g. anti-smoking campaigns, food-distribution programs), rather than effects of removing the outcomes targeted by those actions (e.g. smoking, cancer, malnutrition). Only rarely will the two effects coincide. Focusing on the outcome removal presents a grossly overoptimistic picture of what can actually be accomplished, since the latter is determined by what feasible interventions are available. Focusing on outcome removal has the potential of diverting resources away from where it will do the most good – outcomes with feasible and effective preventives – toward outcomes that, while more common and costly, have less hope of successful and economical prevention. Finally, a focus on outcome removal diverts attention from assessing and comparing the full impacts of interventions. For example, even partial reduction in tobacco use will result in a broad spectrum of outcome prevention, from heart disease to lung cancer, whereas an effective treatment for lung cancer would only reduce the burden from that disease while raising the burden from tobacco-related risks.

The preceding consideration raises another point: Because any action will have multiple consequences, a thorough analysis must consider outcomes in a *multivariate *framework that accounts for the multiple effects of actions and the competition among various outcomes. This multivariate perspective raises serious questions about the value of univariate summaries, which will be taken up after the next section.

## Are socioeconomic indicators causes?

The theory outlined above is strictly a theory of effects of *actions *or *interventions*. It does not formalize all ordinary-language or intuitive uses of the words "cause" and "effect". Two naïve extreme reactions to this limitation have been common: one that denies it is meaningful to talk of causes that are not actions and so restricts "causes" to interventions [33], and one that rejects counterfactual theory outright (see the discussion of Maldonado and Greenland [23]). But two types of constructive reactions have also appeared. The first type generalizes the theory to encompass nonactions as causes, a prime example being the many-worlds theory [5]. While this generalization may best capture ordinary language, it is very controversial and not suitably operationalized for everyday use.

The second constructive response accepts the limitations of the restricted theory and instead seeks to identify potential actions within ordinary events. This approach recognizes that certain "causes" are best treated as intermediate outcomes; one then traces the etiology of such "causes" back to events with intervention potential, or else treats such "causes" as conditioning events and searches for actions that modify or prevent the ultimate outcomes. Earthquakes, which cause extensive destruction, provide neutral examples of unmodifiable causes. An earthquake, *y*_1_, is the outcome of a long and complex chain of events with little intervention potential under today's technology. Perhaps someday we will be capable of interventions that lead to dissipation of crustal stresses with less ensuing damage. But for now, mere prediction would be a major achievement and would facilitate actions to prevent damage when an earthquake occurs. An example of such an action is the enforcement of strict building codes in earthquake-prone regions.

Less neutral examples are provided by common measures of education, such as the classification "No high-school diploma", "High-school diploma", "Some college, no degree", "Two-year degree", "Four-year degree" and "Graduate degree". People believe that education leads to more income and health. But how well do differences in the observed education measure predict the effects of real interventions such as affirmative action, public-school improvements, or scholarship programs? For policy purposes, it is the implementation and evaluation of such programs that matter; the ensuing changes in education measures are only intermediates between the programs and the ultimate goals of improved social, economic and health outcomes.

The value of restricting counterfactual models to interventions is that it forces us to explain observed *associations *between risk factors and health as *outcomes *of potentially changeable events. Consider a highly charged example, "race", which when measured as "white" vs. "black" is strongly associated with many health events. People talk of race as a "cause". But to do something about racial disparities in health outcomes (which is to say, to eliminate the observed association of race and health), we must explain their origin in terms of changeable causes, such as disparities in school funding, availability of individual college funding, prevalence of racist attitudes, etc. Finding feasible interventions and estimating their costs and benefits is required to address observed disparities; asserting or denying that "race" is a cause does not help this endeavor.

## Should different outcomes be summarized in a single number?

Two distinct connotations of summary measure appear extant: the first and most common presumes that the measure summarizes a single outcome variable with a single number. Classic examples include the mortality rate and the life expectancy. The second connotation, largely confined to statistics and physical sciences, allows a summary to be a vector, that is, an ordered list of numbers that summarize different dimensions of a system. An example of such a *multidimensional *or *multivariate *population summary would be the list containing life expectancy, health expectancy, health gap and the proportions of deaths due to various causes (e.g. starvation, violence, infectious disease, heart disease, stroke, cancer).

It should first be noted that all the earlier concepts and discussion apply equally to any action or outcome, whether unidimensional or multidimensional. In particular, the potential outcomes, *Y*(*x*_*j*_), may represent outcome vectors and the alternative actions, *x*_0_, *x*_1_, etc., may also be vectors; for example, *x*_0 _could specify that 30%, 40% and 30% of a fixed budget be allocated to family planning, sanitation and medical supplies, respectively, and *x*_1 _specifies a different allocation scheme. The chief problem in expanding to the multidimensional perspective is the limited number of dimensions that the human mind can contemplate at once. Because that limitation is a key motive for summarization, it is essential to keep track of what is lost in the dimensionality reduction that defines summarization. It also is essential to keep track of the *values *that influence (or should influence) what is kept and what is lost.

Summary measures of population health serve no good purpose when they strongly confound valuations, which vary by individual preference, culture, etc., with measures of occurrence and effect (which are presumably matters of scientific fact, albeit subject to uncertainty). For example, many individuals, in continuing to smoke, explain their behavior as stemming from a conscious preference to die sooner from cardiovascular disease or cancer than survive until mental or neurological deficit is nearly inevitable. For such individuals, measures such as healthy years of life lost due to smoking represent a conflation of someone else's values with the factual risks of smoking, because that summary ignores preferences among various morbidity and mortality outcomes affected by smoking. To give the individual the information necessary for personal choice, we must supply a multidimensional summary that includes lifetime risks of different diseases.

Moving to the societal level, healthy years of life lost due to smoking not only neglects the differences in resource allocation that must exist between present (actual) society and a counterfactual tobacco-free society, it also neglects differences in absolute and proportional morbidity and mortality with and without tobacco use. This neglect is addressed by measures of the economic cost of tobacco use, and by absolute and proportional morbidity and mortality comparisons. By providing all these measures, we shift to a multidimensional summary of tobacco impact.

My intention in raising these issues is not to offer a solution to a specific summarization problem. Rather, it is to remind those facing a choice among measures that candidates need not (and, for policy purposes, should not) be limited to unidimensional summaries. While our ability to think in several dimensions is limited, it can be improved with practice. That practice has proven crucial in attacking problems in physics and engineering, and there is no reason to suppose it is less important in tackling more complex social policy issues. In instances in which many different people must make informed choices based on the same scientific data, but with different values, multidimensional measures are essential if we are to provide each person and each executive body with sufficient information for rational choice.

## Conflicts of interest

The author(s) declare that they have no competing interests.
